# The MIPAM trial – motivational interviewing and physical activity monitoring to enhance the daily level of physical activity among older adults – a randomized controlled trial

**DOI:** 10.1186/s11556-021-00269-7

**Published:** 2021-07-02

**Authors:** Rasmus Tolstrup Larsen, Christoffer Bruun Korfitsen, Camilla Keller, Jan Christensen, Henning Boje Andersen, Carsten Juhl, Henning Langberg

**Affiliations:** 1 Section of Social Medicine, Department of Public Health, University of Copenhagen, Copenhagen, Denmark; 2 Department of Occupational- and Physiotherapy, Copenhagen University Hospital, Rigshospitalet, Copenhagen, Denmark; 3 Musculoskeletal Statistics Unit, The Parker Institute, Bispebjerg and Frederiksberg Hospital, Frederiksberg, Denmark; 4 Research Governance, Evaluation & Communication, Danish Cancer Society Research Center, Copenhagen, Denmark; 5 Department of Technology, Technical University of Denmark, Management and Economics, Lyngby, Denmark; 6 Department of Sports Science and Clinical Biomechanics, University of Southern Denmark, Odense, Denmark; 7 Department of Physiotherapy and Occupational Therapy, Copenhagen University Hospital, Herlev and Gentofte, Denmark; 8 Section of Health Services Research, Department of Public Health, University of Copenhagen, Copenhagen, Denmark

**Keywords:** Older adults, Elderly, Community-dwelling, Health technology, Physical activity, Monitoring, Motivational interviewing

## Abstract

**Background:**

One in four older adults in Denmark and almost half of the very old above 75 do not meet the World Health Organization’s recommendations for a minimum of physical activity (PA). A cost-efficient and effective way to increase focus on and motivation for daily walking might be to use Physical Activity Monitors (PAMs) in combination with behavioural change intervention. Thus, the objective of this randomized controlled study was to investigate the effect of Motivational Interviewing (MI) as an add-on intervention to a PAM-based intervention measured in community-dwelling older adults.

**Methods:**

This two-arm parallel group randomized controlled effectiveness trial compared a 12-weeks PAM-based intervention with additional MI (PAM+MI group) with a PAM-based intervention alone (PAM group). The primary outcome, average daily step count, was analysed with a linear regression model, adjusted for sex and baseline daily step count. Following the intention-to-treat principle, multiple imputation based on baseline step count, sex and age was performed.

**Results:**

In total, 38 participants were randomized to the PAM intervention and 32 to the PAM+MI intervention arm. During the intervention period, PAM+MI participants walked on average 909 more steps per day than PAM participants, however insignificant (95%CI: − 71; 1889) and reported 2.3 points less on the UCLA Loneliness Scale (95%CI: − 4.5; − 1.24).

**Conclusion:**

The use of MI, in addition to a PAM-based intervention among older adults in PA promoting interventions hold a potential clinically relevant effect on physical activity and should thus be investigated further with adequately powered RCTs.

**Trial registration:**

This study was pre-registered in the clinicaltrials.gov database with identifier: NCT03906162.

## Introduction

### Background and objectives

More than 50% of the European older adults are insufficiently physically active [1]. Higher levels of physical activity (PA) among older adults are associated with positive health-related outcomes, including lower levels of frailty [2] and lower levels of all-cause mortality [3]. Furthermore, inactivity among [1] older adults are associated with higher levels of non-communicable diseases, lower functional health, higher risk of depression and cognitive decline [4–6]. Thus, physical inactivity is one of the leading causes of major non- communicable diseases [7]. Furthermore, strong evidence also exists on the positive effects of PA on several chronic diseases including dementia, type 2 diabetes, coronary heart disease, chronic obstructive pulmonary disease, osteoarthritis and several cancers [8].

The *Global Action Plan on Physical Activity “More active People for a healthier World”* published by the WHO in 2018 states: *“global progress to increase physical activity has been slow, largely due to lack of awareness and investment”* [9]. Especially in older adults, easy access to effective PA programs can benefit societies by allowing older adults to maintain an active life and independent living [9]. As walking has been shown to be the most frequent PA modality among older adults [10] and daily step counts to be highly associated with all-cause mortality and cardiovascular disease-morbidity [11], large scale PA programs should include focus on increasing the level of walking in exercising and the amount of walking in ambulant activities.

A cost-efficient way to increase focus on and motivation for a higher level of daily walking is to use Physical Activity Monitors (PAMs) in PA interventions among older adults. A recent systematic review and meta-analysis concluded that the use of feedback from PAMs among older adults was safe, feasible and moderately effective, equivalent to an additional 1300 daily steps, in increasing the daily level of PA [12, 13]. The Internet-Of-Things and wearables in medicine are here to stay [14, 15], and future studies should not investigate effectiveness from the PAMs themselves, but use active comparisons to clarify how Behaviour Change Theories (BCTs) can support wearable devices and self-monitoring of behaviour [12, 13, 16, 17].

Self-monitoring, goal setting, action planning, information about behaviour-health links and the consequences of inactivity are important BCTs in PA-interventions [17–21]. Motivational Interviewing (MI) guides the participants using empathic listening, self-reflection and counselling [22], and aims to facilitate positive behavioural change through increased motivation and increased self-efficacy [23, 24]. MI alone has been shown to be short-term effective in increasing PA among older adults with heart failure [25] and hip fracture [26]. Furthermore, older adults have found the combination of MI and PAM-interventions acceptable [27].

While passive comparisons with PAM-based interventions are no longer needed, clarification on the effectiveness of PAM-based interventions in combination with BCT-interventions is needed [12, 13]. Thus, the objective of this study was to investigate the short-term effect of MI as an add-on intervention to a PAM-based intervention on average daily step count in community-dwelling older adults.

## Methods

### Trial design

The MIPAM trial was conducted as a 12-week, investigator-blinded, two-arm parallel-group, superiority randomized controlled effectiveness trial. This manuscript has been reported according to the CONsolidated Standards of Reporting Trials (CONSORT) 2010 guideline [28]. The allocation ratio between the groups was 1:1 and the only changes to the study protocol [29] was the inability in reaching the desired sample size in the available time period. The methods of this study are described in detail in the study protocol [29].

#### Ethics

The National Committee on Health Research Ethics informed the authors that the trial, being a non-invasive intervention, is not subject to the Danish laws on research ethics (Journal-nr.:18004960). The plan for managing personal and health information of the trial was approved by The Danish Data Protection Agency (Reference number: 514–0268/18–3000). Prior to agreeing and signing the consent survey, the participants received written information about the study. Informed consent from the participants was collected electronically before filling out the baseline questionnaire.

### Participants

Participants were considered eligible for inclusion if they: 1) were retired from the labour market and community-dwelling, 2) were at least 70 years old by the day of enrolling the trial, 3) owned a smartphone or tablet able to install the *Garmin Connect application,* 4) had an active e-mail address 5) were able to fulfil the electronic study survey, and 6) had hearing abilities sufficient to receive oral information about the study and to receive a telephone-based MI intervention. The retirement age in Denmark is currently 65.5 years and is gradually increasing. The age criterion of 70 years was used to avoid including participants between 65 and 70 years who are still fully or partially employed and thus to increase generalizability.

Participants were excluded, if the: 1) had cognitive impairment or mild to severe dementia, 2) were undergoing active chemotherapy or palliative care for cancer, or 3) had a major mobility impairment preventing them from walking.

### Interventions

The PAM group received a PAM-based PA promoting intervention and the PAM+MI group received the PAM-based PA promoting intervention and an MI-intervention as an add-on intervention.

#### Physical activity monitor intervention (PAM)

Participants received a PAM for everyday use in the intervention period and a pamphlet with the national recommendations on PA in aging populations. The specific PAM used in this study is the hip-worn Garmin Vivofit 3 device linked to a pre-specified Garmin Connect account set up with an automatically adjusting daily goal-setting. The participants were asked to wear the PAM for all waking hours, except when bathing, every day for the 12-week intervention period. Participants who experienced installation difficulties received telephone support from the research team not including the blinded primary investigator (RTL).

#### Physical activity monitor intervention plus motivational interviewing (PAM+MI)

The experimental intervention consisted of the PAM intervention in combination with an MI intervention. During the 12-week intervention period, the participants were scheduled to receive seven telephone calls from trained and certified MI-counsellors in intervention week 1, 2, 3, 5, 7, 9 and 12. The MI-intervention was person-centred and participants were guided with self-reflective counselling and received feedback on their health behaviours in relation to the national recommendations [22]. The Social Cognitive Theory and The Transtheoretical Mode were the theoretical frameworks that guided the intervention content to each individual [30, 31]. Self-efficacy and outcome expectations are key constructs and are, among other factors, significant predictors of PA behaviours [31]. Self-efficacy, in this setting for exercise, was operationalized by facilitating confidence when facing barriers to PA, self-monitoring including behavioural goal setting and action planning. Outcome expectancies was operationalized by providing information about behaviour-health link, providing information about consequence and discussion of benefits of and barriers to health behavioural change, which should lead to increased perception of benefits and decreased perception of barriers. Social support was operationalized by identification of supports for maintenance of health behavioural change, and specific goal setting for using supports, which should lead to increase level of support for the participant’s health behavioural change.

In this study, participants in the PAM+MI group were encouraged to use a variety of significant supports including family and friends, as well as neighbourhood and specific community resources (e.g., walking groups proposed by the MI-counsellor).

##### Fidelity

The project MI counsellors were physiotherapists with additional training and education in the MI approach to telephone-based health behaviour counselling. During the study, with participants’ verbal consent, telephone MI sessions were audiotaped on a regular basis to ensure fidelity of intervention delivery and to provide counsellor feedback. Based on a review of these recordings a random segment of 20 min was selected for rating with the Motivational Interviewing Treatment Integrity Scale version 4 (MITI 4) [32], by two independent coders. The MITI 4 is a reliable measure of proficiency in MI practice as defined by Moyers et al. [32]. The MITI 4 consists of four global ratings (Cultivating Change talk, Softening Sustain Talk, Partnership, and Empathy), which are scored on a Likert-type scale from 1 (low) to 5 (high), and 10 individual behaviour counts (Questions, Simple Reflections, Complex Reflections, Persuade with Permission, Giving Information, Affirmations, Emphasize Autonomy, Seeking Collaboration, Persuade and Confront), which are counted within the time frame of the interview [32]. The MI-coders individually coded and reached consensus on MI-behaviour. A median global score in each domain of 4 and a Reflection to Question ratio of > 1 were considered adequate MI proficiency.

### Outcomes

#### Primary outcome measure

The average number of steps per day throughout the 12-week intervention period, measured daily and objectively by the hip-worn Garmin Vivofit 3 tri-axial accelerometer, was the primary study outcome. The Garmin Vivofit 3 has been validated along with three other monitors and the hip-worn PAMs were found to be superior to wrist-worn PAMs in terms of measurement properties among older adults with and without rollators [33].

#### Secondary outcome measures

Secondary outcome measures included self-reported information from the participants on PA, health-related quality of life, loneliness, self-efficacy for exercise, outcome expectancy for exercise, and social relations. According to the protocol the categories MVPA, walking time and sedentary time were estimated with *The International Physical Activity Questionnaire-Short Form (*IPAQ-SF*)* [34–37], MVPA was estimated with *The Nordic Physical Activity Questionnaire short (*NPAQ-short*)* [38, 39], the HRQoL score (EQVAS) was estimated with *The EuroQol-5 Domain (*EQ-5D-5L*) Quality of life questionnaire* [40–43], the total score was estimated with *The UCLA Loneliness Scale* [44, 45] to measure loneliness*,* the sum score was from *the Self-Efficacy for Exercise (SEE-DK)* [46] to used to measure self-efficacy*,* and the sum score was from *the Outcome Expectancy for Exercise-2 (OEE2-DK)* [47] to used to measure outcome-expectancy. Secondary outcome measures are described in greater detail in the study protocol [29]. *The Copenhagen Social Relations Questionnaire* (CRSQ) [48, 49] was used only to inform the MI-counsellors and to determine whether the participants lived alone.

All secondary outcomes were collected at baseline and at post-intervention. The baseline measurement took place before randomization and thus before the PAM+MI group received their first motivational interview; the post-intervention questionnaire was distributed immediately after the 12-weeks of intervention.

### Sample size

To show a moderate effect difference (0.5*standard deviation between group difference) with 80% power and a 0.05 significance level, 128 completed participants were needed. To account for attrition, a 20% dropout was expected and thus, 154 participants (77 in each group) were needed to be allocated to each of the two groups.

### Randomization

Participants were randomly assigned to either the intervention or the PAM group, with a 1:1 allocation ratio. Eligible participants who completed the baseline period of 1 week were randomized in blocks of minimum four participants, stratified on sex and average daily baseline step count for the baseline period. STATA statistical software was used to conduct the stratified randomization. Allocation was concealed for the primary investigator. One investigator (JC) was responsible for the randomization process and had no role in the recruitment of participants nor in the statistical analyses.

### Blinding

The primary investigator (RTL), who was responsible for analyses and data-management, was blinded for participant allocation until the last participant completed the post-intervention questionnaire. As the secondary outcome measures are self-reported, outcome assessor cannot be considered blinded. Due to the nature of the intervention neither participants nor physiotherapists conducting the motivational interviews could be blinded to allocation.

### Data collection and management

Information about data collection management can be found in the study protocol. No deviations from the protocol occurred on this matter [29].

### Statistical methods

Distributions of continuous data was evaluated by inspecting Quantile-Quantile plots of the standardized residuals and histograms with normal distribution curves. Continuous data with normal distributions was analysed with parametrical statistics and summarized with means and standard deviations. Continuous data without normal distribution was analysed as ordinal data with non-parametrical statistics and summarized with medians and interquartile ranges. Categorical or binary data were summarized with frequencies and percentage of total.

The primary outcome, average daily step count, was analysed according to the intention-to-treat (ITT) principle with a linear regression model investigating the between-group differences, adjusted for sex and baseline daily step count. It was chosen to adjust for baseline daily step count to increase the generalizability of the results if any imbalance should have been present after the randomization process. Furthermore, it was chosen to adjust the analyses for sex as differences between men and women have been reported on PA [50], HRQoL [51], loneliness [52]. To adhere to the ITT principle and the effectiveness design, Gaussian normal regression method with predictive mean matching was used to impute missing values (multiple imputation based on baseline step count, sex and age) where less than 7 days of step counts were available for the intervention period. We used 5 imputations as only point estimates were of interest and the amount of missing data was assumed to be low to moderate [53]. The missingness of the step count data was assumed to be missing at random, where any systematic differences could be explained by other observed data [54]. More specifically, the missingness of step count data was assumed to be explained by the age and sex of the participants as both have been reported as predictors of digital literacy [55]. Furthermore, the missingness of daily step count was also assumed to be explained by the level of physical activity. The same procedure was used to analyse between group differences on secondary outcomes, as all secondary outcomes were collected from the electronic survey and the missingness thus assumed to be dependent on digital literacy as well. All secondary outcomes were adjusted for baseline score of the specific outcome, baseline daily step count and sex. Harms, as defined in the study protocol [29], were evaluated by calculating the relative risk (RR), separately for serious and non-serious adverse event between the intervention and PAM group [56]. In calculating the average daily step count, days with less than 100 steps were handled as “days of non-wear” and excluded. A post-hoc power calculation was performed with number of participants, effect size of the between group difference from the primary analysis on daily steps and the baseline overall standard deviation on daily steps. Sensitivity analyses on missingness on the primary outcome include unpaired Student’s t-test to compare the age and baseline daily step count of participants with and without imputed data, a Chi-Square test to compare the sex distribution, a linear regression model for analysing the relationship between number of missing days and age, a Wilcoxon-test testing difference in number of missing days between participants with and without adverse events. Furthermore, to validate the multiple imputation method complete-case and last observation carried forward-analyses were conducted.

RStudio version 1.1.463 for Mac OS X was used for all statistical analyses and illustrations [57].

The CRAN ‘mice’ package was used to perform the predictive mean matching multiple imputations and the ‘ggplot2’ package was used to generate a scatterplot with means and error bars for daily steps throughout the intervention and box plots of secondary outcomes. An alpha level on 0.05 was considered the threshold for statistical significance.

## Results

### Participant flow and information on discontinued participants

**Fig. 1:**
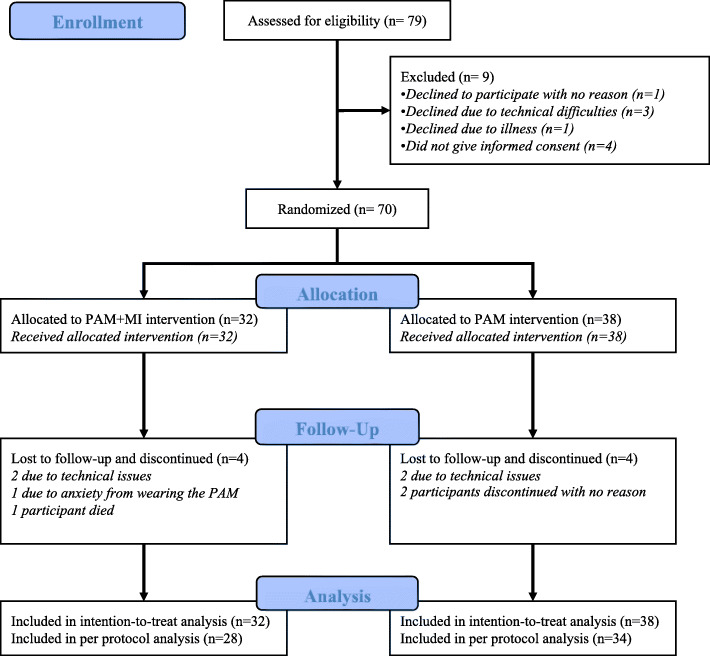
Consort flow diagram

Between May 1, 2019 and January 4, 2020, 79 participants were considered eligible for inclusion and received the trial content. After nine eligible participants refused to participate, 70 participants were included and randomized to one of the two intervention. Of these, 38 were allocated to the PAM intervention arm and 32 to the MIPAM intervention arm. In the PAM intervention arm, 34 participants completed the 12 weeks and four participants discontinued (Fig. 1). In the MIPAM intervention arm, 28 participants completed the 12 weeks and four persons discontinued their participation (Fig. 1). Due to low inclusion rate and insufficient funding to extend the inclusion period, it was decided, to stop inclusion of participants to the trial in January 2020. This resulted in an underpowered trial that did not reach the desired sample size of 128 participants excluding dropouts.

### Baseline data

Socio-demographics and PA characteristics of included participants are reported in Table 1. There were no between-group differences on any variables, except for a higher rate of participants in the PAM+MI group reporting pain (51.6% vs 25.0%). The median age of the participants was 72 years, 28 of 70 participants were male (40.0%), 22 reported to have used a PAM before (32.8%) and the mean baseline daily step count was 5881.

**Table 1:** Socio-demographics and physical activity characteristics of included participants

	Overall (*n* = 70)	PAM group (*n* = 38)	PAM+MI group (*n* = 32)	*p*
**Age, median [IQR]**	72.0 [70.0, 74.0]	71.0 [70.0, 74.3]	73.0 [71.0, 74.0]	0.134
**Sex, n male (%)**	28 (40.0)	16 (42.1)	12 (37.5)	0.613
**BMI, mean (SD)**	27.2 (4.4)	27.3 (4.9)	27.1 (3.9)	0.581
**Education, n (%)**				0.522
No education	1 (1.5)	1 (2.8)	0 (0.0)	
Upper secondary education	11 (16.4)	5 (13.9)	6 (19.4)	
Bachelor’s degree or equivalent tertiary education level	39 (58.2)	21 (58.3)	18 (58.1)	
Master’s degree, equivalent tertiary education level, or above	16 (23.9)	9 (25.0)	7 (22.6)	
**Lives alone, n (%)**	26 (39.4)	13 (36.1)	13 (43.3)	0.507
**Smoking, n (%)**				0.509
Never smoked	33 (49.3)	19 (52.8)	14 (45.2)	
Stopped smoking	29 (43.3)	14 (38.9)	15 (48.4)	
Smokes	5 (7.5)	3 (8.3)	2 (6.5)	
**In pain, n (%)**	25 (37.3)	9 (25.0)	16 (51.6)	0.046
**Long-term chronic disease or disability, n (%)**	33 (49.3)	16 (44.4)	17 (54.8)	0.379
**Limited in usual activities due to disability, health or pain (%)**				0.388
Not limited	35 (52.2)	20 (55.6)	15 (48.4)	
Limited to some extend	26 (38.8)	14 (38.9)	12 (38.7)	
Seriously limited	6 (9.0)	2 (5.6)	4 (12.9)	
**Walking aids (%)**				0.253
None	65 (97.0)	35 (97.2)	30 (96.8)	
Cane	1 (1.5)	0 (0.0)	1 (3.2)	
Rollator	1 (1.5)	1 (2.8)	0 (0.0)	
**% of total activity from walking, median [IQR]**	69.5 [30.8, 80.0]	64.0 [20.0, 80.0]	69.5 [40.0, 79.5]	0.363
**Wants to be more physically active, n (%)**				0.259
Yes	56 (83.6)	28 (77.8)	28 (90.3)	
No	3 (4.5)	2 (5.6)	1 (3.2)	
Do not know	8 (11.9)	6 (16.7)	2 (6.5)	
**Have used or uses physical activity monitor, n (%)**	22 (32.8)	12 (33.3)	10 (32.3)	0.997
**UCLA Loneliness Scale Sum, mean (SD)**	32.9 (8.6)	33.5 (9.5)	32.3 (7.5)	0.399
**EQ-5D-5L**				
Problems with mobility, n (%)	27 (40.1)	13 (36.1)	14 (46.7)	0.373
Problems with self-care, n (%)	4 (6.1)	2 (5.6)	2 (6.7)	0.995
Problems with usual activities, n (%)	19 (28.8)	9 (25.0)	10 (33.3)	0.442
Problems with pain and discomfort, n (%)	43 (65.2)	20 (55.6)	23 (76.7)	0.087
Problems with anxiety and depression, n (%)	13 (19.7)	7 (19.4)	6 (20.0)	0.995
EQ Visual Analogue Scale, median [IQR]	80.0 [70.0, 90.0]	85.0 [70.0, 90.0]	80.0 [70.0, 90.0]	0.438
**Outcome Expectancy for Exercise-2 Scale Sum, mean (SD)**	51.6 (6.9)	50.3 (7.27)	53.1 (6.1)	0.074
**Self-Efficacy for Exercise Scale Sum, mean (SD)**	60.5 (19.8)	59.4 (20.15)	61.8 (20.0)	0.442
**Baseline steps per day, mean (SD)**	5881 (2948)	6029 (3009)	5705 (2913)	0.649
**International Physical Activity Questionnaire Short Form**				
Minutes of vigorous activity per day, median [IQR]	0.0 [0.0, 24.1]	0.0 [0.0, 19.3]	0.0 [0.0, 24.1]	0.581
Minutes of moderate activity per day, median [IQR]	0.0 [0.0, 24.1]	0.0 [0.0, 19.3]	0.0 [0.0, 24.1]	0.581
Minutes of MVPA per day, median [IQR]	0.0 [0.0, 48.2]	0.0 [0.0, 38.6]	0.0 [0.0, 48.2]	0.581
Minutes of walking activity per day, median [IQR]	27.9 [0.0, 327.9]	23.6 [0.0, 422.1]	31.1 [0.0, 214.3]	0.643
MET-minutes per day, median [IQR]	316.9 [63.3, 1386.0]	394.1 [40.9, 1432.0]	254.6 [102.5, 850.5]	0.570
Minutes of sedentary time per day, median [IQR]	303.0 [210.0, 480.0]	303.0 [240.0, 435.0]	316.0 [202.5, 480.8]	0.676
**IPAQ physical activity categories, n (%)**				0.690
High	18 (27.3)	10 (27.8)	8 (26.7)	
Low	26 (39.4)	14 (38.9)	12 (40.0)	
Moderate	22 (33.3)	12 (33.3)	10 (33.3)	
**Nordic Physical Activity Questionnaire short**				
Minutes of MVPA per day, median [IQR]	30.0 [12.9, 68.6]	38.6 [8.2, 82.5]	25.7 [17.5, 46.1]	0.227
Minutes of moderate activity per day, median [IQR]	11.4 [0.0, 24.6]	7.9 [0.0, 34.3]	12.1 [0.0, 19.8]	0.651
Minutes of vigorous activity per day, median [IQR]	17.1 [0.0, 34.3]	19.3 [0.0, 58.4]	13.6 [0.0, 21.4]	0.225
**NPAQ physical activity categories, n (%)**				0.231
Inactive	8 (12.1)	4 (11.1)	4 (13.3)	
Insufficiently physically active	10 (15.2)	7 (19.4)	3 (10.0)	
Sufficiently physically active	12 (18.2)	4 (11.1)	8 (26.7)	
Optimally physically active	36 (54.5)	21 (58.3)	15 (50.0)	
**NPAQ compliance with WHO recommendations, n (%)**	48 (72.7)	25 (69.4)	23 (76.7)	0.490

*BMI* Body Mass Index, *PAM* Physical Activity Monitor, *EQ-5D* EuroQol Research Foundation Five Domains, *UCLA* University of California Los Angeles, *OEE* Outcome Expectancy for Exercise, *SEE* Self Efficacy for Exercise, *IPAQ-SF* International Physical Activity Questionnaire-Short Form, *NPAQ* Nordic Physical Activity Questionnaire-Short Form, *MVPA* Moderate to Vigorous Physical Activity, *SD* Standard deviation, *IQR* Interquartile range, *IPAQ-SF* International Physical Activity Questionnaire-Short Form, *NPAQ* Nordic Physical Activity Questionnaire-Short Form, *MVPA* Moderate to Vigorous Physical Activity. Test for between-group difference in normal distributed continuous variables (BMI, UCLA Loneliness Scale Sum Score, Outcome Expectancy for Exercise-2 Scale Sum Score, Self-Efficacy for Exercise Sum Score and Baseline Daily Steps) were performed with unpaired t-test, test for between group difference in non-normal distributed continuous (age, % of total activity from walking, EQ Visual Analogue Scale, all IPAQ and NPAQ scores) variables were performed with Mann-Whitney U test, test for between group difference in categorical or binary variables with Chi2 test, *p*-values ≤0.05 are considered significant

### Numbers analysed

The median days of missing PA data during the 12 weeks of intervention was 6 [IQR: 1, 32] days in the PAM group and 4.5 [IQR: 0.75, 26] in the PAM+MI group. Data for four participants were imputed for average daily steps. Data for six participants were imputed for IPAQ-SF MVPA and minutes of sedentary time per day, NPAQ-Short MVPA minutes per day, EQ-VAS, UCLA Loneliness Scale Sum Score, and SEE-DK Sum Score. Data for seven participants were imputed for IPAQ-Short minutes of walking per day and OEE2-DK Sum.

### Outcomes and estimation

For the primary outcome, the PAM+MI group increased by 909 steps daily throughout the intervention period compared to the PAM group, but insignificantly (95%CI: − 71; 1889). For the secondary outcomes, the participants in the PAM+MI group reported 2.3 UCLA Loneliness Scale Sum Score points less compared to the PAM group (95%CI: − 4.5; − 1.2). No relevant or significant differences were found in the other secondary outcomes (Table 2).

**Table 2:** Results from multiple regression models on outcomes

Outcome	Post-intervention scores		Adjusted between group difference from multiple regression model		
	PAM group (***n*** = 38) mean (95%CI)	PAM + MI group (***n*** = 32) mean (95%CI)	Between group difference	95%CI	***p***
**Average daily steps**	5837 (4932; 6742)	6492 (5472; 7513)	909	(−71; 1889)	0.07
**IPAQ-SF**					
MVPA minutes per day	53.9 (15.3; 92.5)	34.4 (5.2; 63.6)	−0.2	(−46.3; 45.8)	0.992
Minutes of walking per day	149.2 (59.1; 239.3)	218.5 (111.5; 325.5)	78.1	(−6.1; 217.3)	0.266
Minutes of sedentary time per day	358.5 (303.6; 413.4)	335.0 (273.0; 397.0)	−40.3	(−102.8; 22.1)	0.201
**NPAQ-Short**					
MVPA minutes per day	72.5 (41.0; 104.0)	66.6 (40.1; 93.1)	−3.8	(−45.3; 37.7)	0.856
**EQ-VAS**	80.6 (76.0; 85.1)	81.6 (78.2; 85.1)	2.9	(−1.9; 7.7)	0.227
**UCLA Loneliness Scale Sum Score**	32.8 (29.6; 36.0)	30.2 (27.4; 33.0)	−2.3	(−4.5; −1.2)	0.04
**Self-Efficacy for Exercise Sum Score**	52.5 (45.9; 59.1)	55.3 (45.9; 60.4)	3.5	(−4.3; 11.2)	0.375
**Outcome Expectancy for Exercise-2 Sum Score**	51.3 (48.5; 54.2)	53.2 (50.5; 56.0)	2.0	(−2.0; 6.0)	0.320

*Abbreviations*: *IPAQ-SF* International Physical Activity Questionnaire Short Form, *NPAQ-Short* Nordic Physical Activity Questionnaire Short, *EQ-VAS* EuroQol Visual Analogue Scale, *UCLA* University of California, Los Angeles, Data for four participants were imputed for average daily steps. Data for six participants was imputed for IPAQ-SF MVPA and minutes of sedentary time per day, NPAQ-Short MVPA minutes per day, EQ-VAS, UCLA Loneliness Scale Sum Score, and SEE Sum Score. Data for seven participants was imputed for IPAQ-Short minutes of walking per day and OEE-2 Sum. End point scores are unadjusted. Primary analysis is the multiple linear regression model adjusted for baseline score, baseline steps, age and sex. Coefficients > 0 means higher value in the PAM+MI group. Negative coefficients for IPAQ-SF Sedentary Time and UCLA Loneliness Scale Sum Score means less sedentary time and loneliness in the PAM+MI group. *P*-values < 0.05 is considered significant

**Fig. 2:**
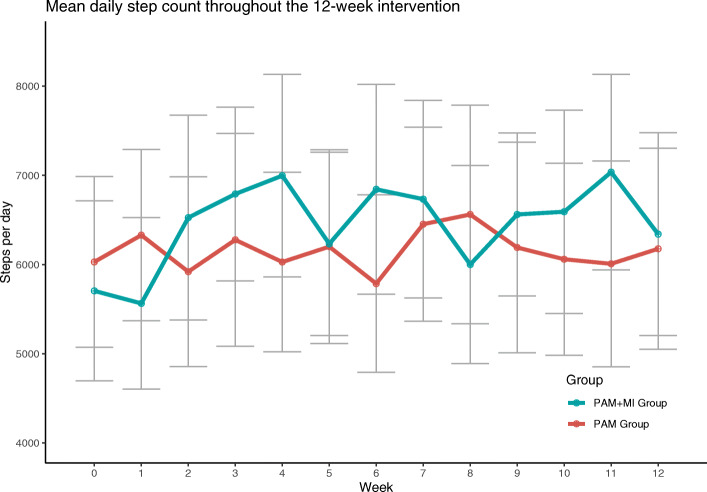
Unadjusted mean daily step counts throughout the 12-week intervention. W0: baseline week. Intervention period: w1 to w12. Circles represent mean values and error bars represent 95% confidence intervals

**Fig. 3:**
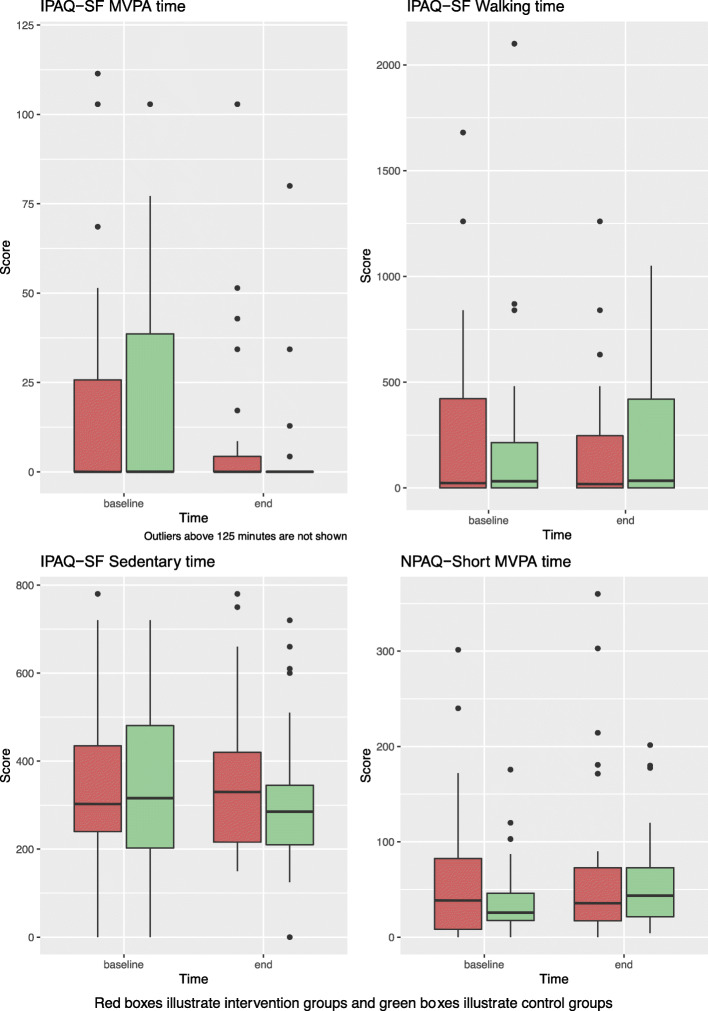
Box plots of secondary self-reported physical activity outcomes at baseline and postintervention endpoint. Red boxes showed the PAM+MI group values and green boxes showed the PAM group values. Thick vertical lines are medians, box size represent interquartile range from 25th to 75th percentile. Notches represent median ± 1.57*IQR/ √*n*, and dots represent samples outside this range. Abbreviations: IPAQ-SF: International Physical Activity Questionnaire Short Form, MVPA: Moderate to vigorous physical activity, NPAQ-Short: Nordic Physical Activity Questionnaire Short. Unimputed data are presented in the boxplot

**Fig. 4:**
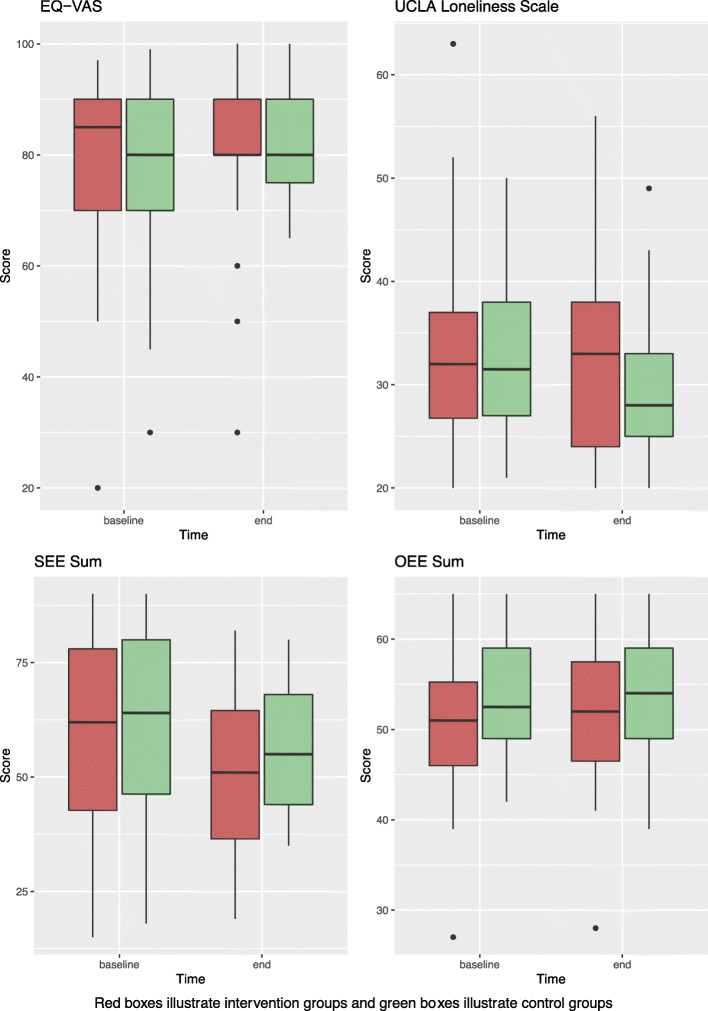
Box plots of secondary self-reported outcomes at baseline and post-intervention. Red boxes showed the PAM+MI group values and green boxes showed the PAM group values. Thick vertical lines are outcome medians, box size represent interquartile range from 25th to 75th percentile Notches represent median ± 1.57*IQR/ √*n*, and dots represent samples outside this range. Abbreviations: EQ-VAS: EuroQol Visual Analogue Scale, UCLA: University of California Los Angeles, SEE: Self-Efficacy for Exercise, OEE: Outcome Expectancy for Exercise-2. Unimputed data are presented in the boxplot

Figure 2 illustrates unadjusted steps per day for the two study arms through the study period. In the Appendix, figure 3 and figure 4 illustrates box plots of other secondary outcomes at baseline and end point for both treatment arms.

#### Fidelity

Each participant in the PAM+MI group was scheduled to receive seven MI calls. Among the 28 complete case PAM+MI group participants, 23 (82.1%) received all seven calls, four (14.3%) received six calls and one participant (3.6%) received four calls. In total, 170 calls with an average length of 18.4 min were delivered to the PAM+MI group. Six MI calls were audiotaped and coded by the two coders using the Motivational Interviewing Treatment Integrity Scale. The median cultivating-change talk global score was 3.5, the median softening sustain talk was 4, the median partnership score was 4, and the median empathy score was 4. The median number of Giving Information was 3.5, the median of Simple Reflections was 3, the median of Questions and Complex Reflections was 7, the median of Affirm and Seek was 1 and 1.5 respectively and the median number of Persuade, Persuade with Permission, Emphasize Autonomy and Confront was 0. The ratio of Reflections to Questions was 1.3.

### Ancillary analyses

The eight participants who discontinued the intervention differed significantly from the complete cases as they were older 78.5 years [IQR: 74.0, 81.5] compared with 72.0 years [IQR: 70.0, 74.0], *p* = 0.035, only female (54.8% female in complete case versus 100% female in discontinued participants, *p* = 0.038), and had a different use of walking aids (one rollator user and no cane users in the complete case versus with a cane user and no rollator users in discontinued participants, *p* = 0.006). No other significant or clinically relevant differences were found on other baseline variables.

A post-hoc power calculation of the primary analysis, showed a level of power on 24.6%. This analysis included 70 participants, an effect size on 909 steps, the standard deviation of the daily step count of 2948 and an alpha level of 0.05.

#### Missingness

The participants with imputed data for PA were all female (*p* = 0.092), on average 8.5 years older (95% CI: 6.6; 10.7) and had a 67 step (95%CI: − 3948; 4081) higher daily step count in the baseline week compared to the participants who did not have their intervention step count imputed. The number of missing days was not dependent on the probability of reporting adverse events, illness or similar. The 56 participants who completed the post-intervention survey without reporting any illness or other, similar adverse events had a median amount of missing days of 5. The eight participants who completed the post-intervention survey and did report an adverse event, illness or similar, had a median amount of missing days of 2.5. A Wilcoxon test revealed no significant difference between the groups, *p* = 0.362. Between-group difference sensitivity analyses without multiple imputation revealed a between-group difference of 889 steps (95%CI: − 99; 1877), *p* = 0.077, for the complete case analysis and 825 steps (95%CI: − 110; 1762), *p* = 0.08, for the intention-to-treat analysis with last observation (baseline week) carried forward.

### Harms

The frequencies of dropouts from the two groups were similar with four dropouts in the PAM+MI group (12.5%) and four dropouts in the PAM group (10.5%). Two participants, both allocated to the PAM+MI group (6.3%), discontinued due to adverse events, as judged by the investigators. One participant died and one participant had increasing anxiety of wearing the PAM triggering existing mental illness. There was no significant between group difference between the proportions of adverse events in the groups (0% in the PAM group versus 6.3% in the PAM+MI group, *p* = 0.400).

## Discussion

To our knowledge, the MIPAM trial is the first study to investigate the effect of adding MI to a PAM-based PA intervention among community-dwelling older adults aged 70 or above. As this study had insufficient power no final conclusions can be drawn about the true effect of the intervention. However, the PAM+MI group walked on average 909 (95%CI: − 71; 1889) steps more per day compared to the PAM group. Even though this finding is non-significant, the confidence interval suggest that MI might possible increase the average level of PA when adding it to a PAM-based PA intervention.

The research team chose objectively measured PA as the primary outcome of interest as the aim of trial was to investigate behaviour change related to PA. However, the real-world interest of clinicians and healthcare workers might not be focused on the PA levels among older adults but on hard outcomes such as disease prevalence and mortality. Thus, daily PA might not be categorized as critical for decision making [58] and the results of this trial cannot be extrapolated to conclude upon the associations between the measured behavioural change and critical outcomes. However, PA levels among older adults are associated with levels of non-communicable diseases, functional health, risk of depression and cognitive function [4–6]. As physical inactivity remains one of the leading causes of major non-communicable diseases [7], daily PA levels serve as a highly relevant construct to measure and as one of the most important surrogate outcomes for critical outcomes among older adults [59, 60]. Evidence suggests that a PA level of 7100 steps per day (if averaged over a week) is enough for older adults to meet WHO recommendations for PA [61]. Additionally, for each increment of 1000 steps per day, the risk of all-cause mortality decreases with 11% even after being adjusted for several confounding factors [62]. In summary, MI might hold the potential of keeping older adults more physically active over a 12-week intervention study, and the difference between the MI plus PAM and the PAM alone group is clinically relevant for older adults.

This trial failed to reach a sample size of 128 participants and consequently should be categorized as underpowered. The post-hoc power calculation revealed a 24.6% power in this specific study, for being able to reject the null hypothesis, when it should be rejected. Thus, the between group difference on 909 (95%CI: − 71.; 1889) steps per day may be an overestimation. However, when inspecting the confidence interval for the primary analysis, the between-group difference lies between 71 steps in favour of the PAM group and 1889 steps in favour of the PAM+MI group. Hence, it seems very plausible that the PAM+MI group had a higher daily step count in the intervention period.

Physical activity is a difficult construct to measure with many considerations about practicality, feasibility and validity [63]. The existing literature on randomized controlled MI-based studies investigating physical activity in older adults uses different measures of physical activity including objectively measurement of subgroups of the study, comparing accelerometer measured baseline weeks with end-point weeks, recall questionnaires and physical activity diaries [25–27, 64, 65]. Physical activity is a behaviour that should be measured consequently over the period of interest, especially in intervention research as the changes and between group differences might occur during the trial, and not before and after the trial. This problem also exists in the observational literature linking disease, morbidity and mortality with physical activity in older adults or in general [3, 62, 66, 67], and even though these observational studies includes large samples which leads to precise estimates, the Hawthorne effect, defined as immediate behavioural change expected from research participation, cannot be ruled out and might impose different types of bias [68]. Especially in moderately sized experimental behavioural change studies, physical activity should be measured consecutively and conclusions should be drawn on accumulated or average physical activity and not on point estimates. When inspecting the means of Fig. 2, it comes clear that a high degree of variability exists from week to week and if one of these weeks were used as end-point outcome alone, different conclusions could be drawn. However, the variability could also be explained by the large variation in the data and the relatively few samples, but as a methodological consideration it comes clear that behavioural change studies should include consecutively measured constructs, which is a strength of this study.

The secondary outcomes assessed in this study include self-reported PA, health related quality of life, loneliness, self-efficacy for exercise and outcome-expectancy for exercise. Besides UCLA Loneliness Scale, none of the secondary outcomes were significantly different between the groups at endpoint. The UCLA Loneliness Scale Sum Score was 2.3 points (95%CI -4.5; − 1.24) lower in the PAM+MI group. Because the literature lacks a minimal clinically important differences on the UCLA Loneliness scale among older adults, this can also be interpreted as a small to moderate effect size (Cohens d: 0.38) [69]. However, this can easily be explained by the nature of the intervention, as MI uses active empathic listening, self-reflection and counselling [22], which naturally affects some of the items used in the UCLA Loneliness scale. Furthermore, some of the difference can also be explained by a small insignificant difference between the groups at baseline and extrapolation of these results should not be done on this secondary outcome, but on future well-powered studies using Loneliness as the primary outcome. In summary, with all the limitations to the finding on loneliness, it is still a relevant difference, and as loneliness has been reported to affect self-reported health and PA negatively this finding might be associated with a higher activity level in the PAM+MI group [70].

To our knowledge, most studies published on MI interventions among older adults targeting PA behaviour directly include intervention lengths from 8 weeks to 6 months [25–27, 65]. Furthermore, a systematic review and meta-analysis reported the median length of PAM-based interventions among older adults to be 12 weeks, ranging from 4 to 52 weeks [12]. Hence, the intervention length on 12 weeks of this study is in line with former studies. It is possible that the exposure to MI was too short to demonstrate an actual effect within the 12 weeks and that the results of this trial only reflects the initial and short-term behavioural changes and thus not the long-term effects. To investigate the long-term effects of this 12-week intervention, 6 and 12-month follow-ups will be conducted as it is hypothesized that the MI intervention will help the participants develop more effective strategies to ensure long-term adherence to healthy PA behaviour. Even so, the quality of the MI calls was considered adequate as our results on global ratings of the content ranged from 3.5 to 4 out of 5 (where higher scores indicate higher integrity of the content) and the reflection to question ratio was 1.3. As previously described, MI has been reported to be effective on short-term outcomes, but a recently published study with 1742 participants did not report any effect of either group-based or individual-based MI [71]. Thus, MI might be effective in some populations, and not in others.

This study used multiple imputation to adhere to the ITT principle. It was assumed that the missingness was at random, and it was, as expected, partly explained by older age and being female (not significantly). With this small sample, the multiple imputation method and the basis of the imputations can be questioned [54]. However, the number of imputations were relatively small and the last observation carried forward (ITT) sensitivity analysis and the complete-case analysis showed highly similar point estimates and variances. Thus, it would have made no difference to choose another ITT-approach.

### Limitations

This study and the results come with several limitations. Firstly, as previously discussed, the sample size was not large enough to ensure adequate power for this study. Secondly, the inclusion and exclusion criteria for this trial were only ensured by telephone or e-mail by the primary investigator. Thus, it is possible for participants to fall under one or more of the exclusion criteria and still participate in the study, if they (willingly or unwillingly) withheld pertinent information from the primary investigator. Nevertheless, even though this is possible, this potential problem should be balanced between groups, as the randomization occurred after the baseline period. Thirdly, the study participants were not blinded for group allocation and consequently performance bias could have been introduced. This type of problem is common in PA intervention studies, and might cause for an exaggeration of study effects [72]. However, a more recent published meta-analysis of more than 1100 trials reports found no evidence for an average difference in effect sizes between adequately blinded studies and studies that lack blinding of either participants, healthcare providers or outcome assessors [73]. This trial tried to control for this by using an objectively measured primary outcome that neither participants nor healthcare providers could affect.

### Interpretation and reflections from the motivational sessions

Limitations related to the MI-sessions also exists. Reflections from these MI-sessions were not systematically collected and thus should only be used for researchers and health care workers planning to conduct MI among older adults.

Firstly, the first telephone calls were mainly used to form the relationship between the counsellor and the participant and rarely for actual MI-content. Secondly, the participants included in this trial were mainly well-educated, active and resourceful older adults with high levels of health literacy, which might affect the generalizability to the background population of older adults, as previous research has shown that exercise and physical activity adherence are associated with resources such as social support and the ability to understand the benefits of physical activity [74–76].

Lastly, using the Garmin application or navigating the smartphone in general, were frustrating to many participants, however, feedback related to the automatic goal-setting was useful for many. In general, participants were motivated to be committed and pushed to plan more challenging goals, thus these sessions were primarily coaching rather than MI that are normally used among less motivated individuals.

## Conclusion

This RCT found a clinically relevant but insignificant difference of 909 (95%CI: − 71.; 1889) daily steps in favour of the PAM+MI group. The use of MI, in addition to a PAM intervention, among older adults in PA promoting interventions should be investigated further in sufficiently powered RCTs.

## Data Availability

Anonymized data are available upon request.
